# Real-Time Triage, Position, and Documentation (TriPoD) During Medical Response to Major Incidents: Protocol for an Action Research Study

**DOI:** 10.2196/57819

**Published:** 2024-12-19

**Authors:** Monica Rådestad, Torkel Kanfjäll, Veronica Lindström

**Affiliations:** 1 Department of Clinical Science and Education, Södersjukhuset Karolinska Institutet Stockholm Sweden; 2 Capio Saint Göran's Hospital Stockholm Sweden; 3 Samariten Ambulans AB Stockholm Sweden; 4 Department of Nursing Umeå University Umeå Sweden; 5 Division of Ambulance Service, Region Västerbotten Umeå Sweden; 6 Department of Health Promotion Science Sophiahemmet University Stockholm Sweden

**Keywords:** action research, decision support technique, information technology, medical response, major incident, management

## Abstract

**Background:**

There is a need to address the implementation of technological innovation into emergency medical services to facilitate and improve information exchange between prehospital emergency care providers, command centers, and hospitals during major incidents to enable better allocation of resources and minimize loss of life. At present, there is a lack of technology supporting real-time information sharing in managing major incidents to optimize the use of resources available.

**Objective:**

The aim of this protocol is to develop, design, and evaluate information technology innovations for use in medical response to major incidents.

**Methods:**

This study has a qualitative action research design. This research approach is suitable for developing and changing practice in health care settings since it is cyclical in nature and involves development, evaluation, redevelopment, and replanning. The qualitative data collection will include workshops, structured meetings, semistructured interviews, questionnaires, observations, and focus group interviews. This study assesses the use of a digital solution for real-time information sharing by involving 3 groups of indented users: prehospital emergency care personnel, hospital personnel, and designated duty officers with experience and specific knowledge in managing major incidents. This study will explore end users’ experiences and needs, and a digital solution for prehospital and hospital settings will be developed in collaboration with technology producers.

**Results:**

The trial implementation and evaluation phase for this study is from April 2024 to May 2026. Interviews and questionnaires with end users were conducted during the planning phase. We have performed observations in connection with 2 major exercises in April 2024 and November 2024. The outcome of this analysis will form the basis for the design and development of a new information technology system. We aim to complete the observations in training sessions and exercises (phase 3) by September 2025, followed by modification of the technology solutions tested (phase 4) before dissemination in a scientific journal.

**Conclusions:**

This protocol includes several methods for data collection that will form the basis for the design and development process of a digital solution for real-time information sharing to support efficient management in major incidents based on the experiences and requirements of end users. The findings from this study will contribute to the limited research on users’ perspectives and the development of digital solutions for real-time information during major incidents.

**International Registered Report Identifier (IRRID):**

PRR1-10.2196/57819

## Introduction

Information technology plays an important role in the improvement of information exchange during a medical response to a major incident (MI) or disaster [1-6]. However, effective information sharing and transfer between the field and command centers continues to remain a problem [7-9]. The greatest challenge in a high patient surge setting is the coordinated management of limited resources to optimize patient care and save lives [2,7,10-12]. The medical response to an MI is the action taken immediately following the incident to reallocate available resources; mobilize additional resources; and triage, treat, and allocate patients to hospitals for definitive care [13]. In this paper, an MI is defined as “a situation in which available resources are insufficient for the immediate need of medical care” [13].

Inadequate or incorrect flow of information on the resources required and the number of patients involved and their injuries has a negative impact on the management of MIs and disasters. This has been highlighted in several systematic reviews, reports, and inquiries [14-16]. Resource management and logistics problems after an MI are often identified as caused by communication failure and not by the lack of medical resources [17,18]. Resource allocation is based on decisions made at the scene of the incident, at the hospital, and at regional and national command centers. Decision-making under extreme stress is very demanding. In a study by Hugelius et al [19], prehospital emergency care personnel in the emergency medical services (EMS) experienced radio communication to be less supportive than expected in a noisy and chaotic environment.

There is widespread interest in information and communication technologies for the transfer of data in an emergency setting [5,7,8,11,20,21], and many believe that technology has the potential to vastly improve the medical response to MIs [2,4,8,11,18,21,22]. Information technology has been tested in various command situations [1,11], including EMS [11,21,23], at the hospital [11,24-28], in pediatric care [29], and in a military context [30]. Even if information technology has advanced in recent times, there are still barriers to going from a project stage to an operational implementation phase [31]. One challenge or barrier described by Zhang et al [7] relates to the limited ability of prehospital emergency care personnel to communicate when dealing with critically injured patients in the field, as their foremost function is to perform hands-on examinations and treatments on patients. Given the extreme workload in a stressful environment, the prehospital emergency care personnel’s ability to use handheld devices for communication has been perceived as a hindrance to the user [32].

In 2004, Chan et al [11] proposed the use of wireless technology in MIs to track patients, report patient numbers, triage status, and allocate resources by automatic sharing of information between devices. Haverkort et al [25] argue that patient tracking and tracing systems should cover both prehospital and hospital care and that performance of triage, registration, documentation, and tracing functions should be given equal importance.

Prehospital emergency care personnel still rely on radio communications and verbal reports to share and transfer information of the amount of injured and triage category (eg, red, yellow, green, black, possibly blue) from the scene of the incident. This has resulted in delayed and incomplete transfer of information both at the scene of the incident and after admission to hospital [6,7,11,16,25]. Breakdown in communication is often cited as a challenge that information technology can address. A systematic review of patient tracking technologies by Dobson et al [29] identified a lack of studies that were methodologically strong. Furthermore, most studies focus on adopting techniques with little attention paid to reliability and usability, that is, intended users’ perceived usefulness and ease of use [5,7,32,33]. Evidence-based practice must be defined to decrease the risk of poor outcomes [34]. However, according to Zhang et al [7], Chan et al [11], Lee et al [31], and Waterman et al [35], research into the practical implementation of innovations in a complex environment is no easy task. For implementation to be successful and for it to improve the quality of medical response, users must be ready and eager to adopt new technology. Providing real-time data is an area where new technology can excel in MI management [11]. The limitation of information sharing during MIs leads to insufficient communication and common operating pictures at command centers that hinder management decision-making [7]. Chan et al [11] highlight the need for developing support for managing an MI. However, there remains a gap in knowledge regarding the collaborative design and development of supportive technologies with end users as well as their practical application. Therefore, to address the need for improved communication and to make the users eager to adopt new technology, our project aims to co-develop a real-time communication and information system with collaboration with prehospital emergency care professionals, stakeholders, and MI managers. By actively involving users, we aim to create a solution that meets operational needs and facilitates implementation in a complex environment.

A real-time communication and information system for Triage, Position, and Documentation (TriPoD) will be developed, designed, and evaluated in collaboration with intended users. Specifically, the project will develop a seamless, automated information system to support the real-time transfer of data to all health care management levels. The system will include electronic triage tags, mobile devices, a web portal, and patient-tracking devices covering prehospital and in-hospital settings. A key feature of the study project is automatic information transmission through the usage of electronic tags (each tag has a unique ID and is attached to each patient) from the scene of the incident to the hospital and within the hospital, which will provide timely and accurate information visualized on data screens at command centers at different management levels. TriPoD will also permit re-evaluation in real-time to be made, as triage is a dynamic process. At the user level, the systems developed must be easy and intuitive to use and not interfere with the user’s ability to perform optimally [11]. This is why the viewpoints of the intended users will be addressed using cyclic action research [36], where iterative design is an essential part of the methodological approach. Action research focuses on generating knowledge, facilitating action, implementation, and evaluation of context-specific solutions [37]. Therefore, the aim of this study protocol is to develop, design, and evaluate information technology innovations for use in medical response to MIs.

## Methods

### Study Background

This study adopts an action research approach [36], which is a cyclic study design and involves development, evaluation, redevelopment, and replanning. Action research is a suitable method for developing and changing practice in the health care setting [37]. Action research aims to improve practice by involving those directly affected by it in the research process [38] and can be defined as “research strategies that address real world problems in a participatory, collaborative, and cyclical way to produce knowledge and action” [39]. In the subsequent studies, involvement and collaboration will be performed with intended users to identify and solve the most important issues regarding information exchange in the medical response to MIs. By collaborating with the intended users, the researchers’ team will receive information about the current practices and thereby understand what is required to be changed for the better. According to Dinsmore et al [40], the strength of action research is the researcher’s capability to identify and create solutions to practical problems by using different methods such as focus groups interviews and observations. In addition, the design advocates that the action of the researcher is to be a part of the research process where the act itself is used as a tool to generate essential knowledge and then to develop and change the system [41]. In this context, part of the researcher’s task is to identify and listen to intended users to develop a real-time communication and information system that will facilitate management decision-making during an MI. The following research questions will form the basis of the action research:

1. How do we create interoperable robust technology for medical management at different command levels during an MI?
2. How will we capture and share information in chaotic and stressful situations?
3. How should informatics and technology solutions be designed to fit different levels of management in the medical response to MIs?
4. How can information be easily shared between medical teams and command centers to provide a real-time accurate overview, and how should they act based on this information?

The research process is cyclic, where planning, action, observation, and reflection are repeated until a satisfactory result is achieved. The working group in this study consists of academicians, health care professionals from EMS, hospitals, designated duty officers, and information technology producers.

### Study Setting

This study will be conducted from April 2024 to May 2026 in Region Stockholm, which is the largest health care provider in Sweden with 6 emergency hospitals, 3 pediatric emergency departments, 10 local emergency clinics, and 9 minor hospitals. Region Stockholm includes Stockholm County with 26 primary municipalities and a population of 2.4 million inhabitants (>20% of the total Swedish population). In terms of area, Stockholm County is one of the smaller counties in Sweden (approximately 6500 square kilometers).

In Sweden, there are 3 command levels in the event of an MI: national, regional (strategic), and local. The local level includes prehospital and hospital management. The EMS is a pool of regional resources, including agencies, health care providers, personnel, equipment, and facilities, under the command of the regional authority. This study protocol focuses on ambulance and hospital care. In Sweden, the first ambulance personnel will adopt the position of the prehospital incident commander in case of an MI. This position has the mandate to lead and organize the prehospital medical response, distributing prehospital resources and working together with relevant partners such as police and firefighters on-site [19].

The planned studies will involve one of the 6 emergency hospitals serving approximately 420,000 inhabitants. Regardless of levels, all command centers deal with the mobilization and allocation of resources according to expected requirements. Higher levels have the authority to increase the framework of resources available. Effective response requires accurate and timely information from the field to the hospital and within the hospital. In this study protocol and in the subsequent planned studies, the term command will be used to cover both prehospital and hospital MI management. Medical management at each level comprises the incident commander and the chief medical officer.

### Participants

The overall aim has been predetermined and defined by the research team. Data collection started in April 2024 and is ongoing. Intended users are actively involved in the development, design, and evaluation of the technology system developed for information sharing and transfer of real-time data. The Stockholm County EMS organization is involved in the project, and e-triage, patient-tracking, and trace systems will be tested and evaluated in both prehospital and hospital settings. The recruitment of the participants will be strategic and purposive, including personnel with specific knowledge, experience, and wishes related to information technology. A total of 3 participant groups will be included in this study: prehospital emergency care personnel, hospital personnel (including physicians, nurses, and administrators), and designated duty officers. Qualitative studies have no optimal sample size since they depend on the purpose of the study and the richness of data [42]. However, since this study is based on homogenous groups, the sample size will be smaller, and the participants will be recruited until information power is reached. The need for specific experiences and expertise among participants is crucial in our studies and limits the number of participants that can be and need to be included. However, information power will be assessed throughout the research process by using preliminary analyses as recommended by Malterud et al [43].

### Study Design

**Figure 1:**
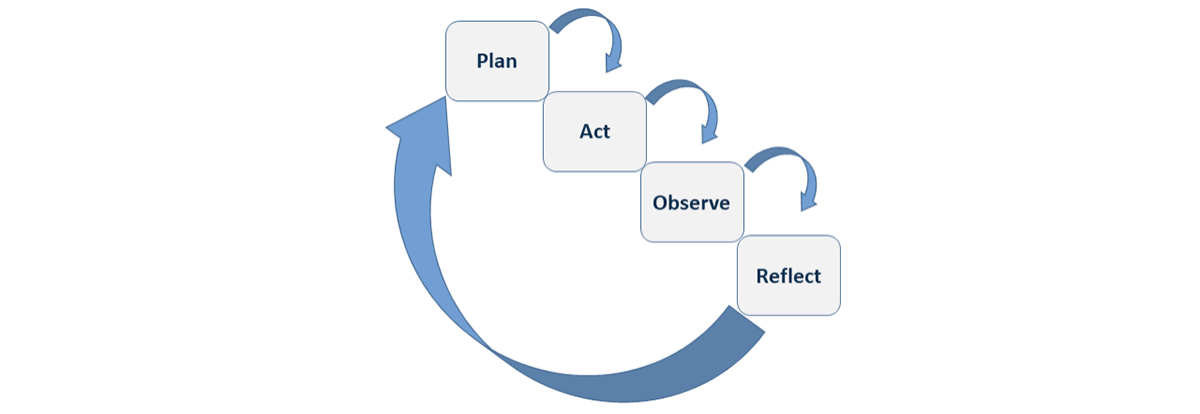
The action research spiral.

This project is designed to stimulate reflection among users through discussion and feedback. Action research is an iterative process that depends on a continuous dialogue between the research team, information technology producers, and intended users [39]. The selected cyclic process comprises 4 iterative phases described by Kemmis et al [36]: (1) planning, (2) action, (3) observation, and (4) reflection (Figure 1). Each phase uses mixed methods to explore end user experiences, communication challenges, and attitudes to new technology.

#### Phase 1: Planning

The starting point has been structured meetings and workshops with different health care professionals, stakeholders, researchers, senior lecturers in EMS, and technology producers (n=10-12) to define the problems to be investigated. Furthermore, previous research has been reviewed to gain an overview and understanding of which real-time communication and information systems have been tested or used. This was done, as changing systems and work processes are one of the fundamental challenges to the digitalization of services in health care. To obtain insight into the current communication methods, the needs and impact of new technology in response to MIs, and the desired way forward presented among prehospital emergency care personnel (n=18), we used questionnaires and interviews for collecting data. Findings have been used to plan for the development of technical and user-friendly aspects of TriPoD. During the planning phase, interviews will also be conducted with 5-10 duty officers and hospital personnel to understand and explore known communication challenges from their perspective when working in the management function during an MI. The interviews will be digitally recorded and transcribed verbatim. Data will be analyzed using content analysis as described by Elo and Kyngäs [44], and to reduce bias, the data analysis will involve multiple researchers. After problem identification and the outcome from the content analysis, a technological innovation for use in the medical response to an MI will be developed and designed, and this will become the starting point for reflection and problem-solving according to action research. This will form the basis of the analysis and will be reflected in the project results.

#### Phase 2: Action

The basic idea of action research is to create participation, dialogue, and reflection in the development and design process as well as to capture user reactions to the innovation technology such as ease of use, utility, and efficiency. In this phase, the developed technical prototypes will be pilot tested in prehospital and hospital settings, and user feedback will be used to take new ideas forward in the technical development process. The specific topic for this data collection is to obtain deeper knowledge from the pilot tests on how to further improve the design of the digital system. Based on the findings from the tests, an assumption is that the users’ opinions and reflections can be applied to the development process of TriPoD. This phase of data collection involves field observations during exercises and has been practiced in 1 exercise with figurants concerning the management of an MI. A qualitative observational design with nonparticipant observers stationed at the incident site at a regional command center and at a hospital command center was used in the early summer of 2024. Observers systematically compared standard procedures and management with TriPoD and made field notes during the test. Trying new ideas and assessing how they function in practice is important in the cyclic process of action research enabling users and project partners to find solutions. A reference group of 8-10 indented users has been established during spring 2024. This is an important phase where users are encouraged to suggest possible changes themselves. In the literature, this phase is described as increasing the likelihood of solution sustainability [37]. It can also enrich the understanding of the specific settings in which the new system will be used.

#### Phase 3: Observation

In this phase, the research team will observe and evaluate the performance and outcomes from phase 2. The TriPoD system must be intuitive and user-friendly, given the stressful and potentially hazardous environments in which it is used. Phase 3 involves observations of current practice and includes (1) creation of test situations in prehospital and hospital settings to simulate real-time patient flow and tracking, (2) assessment of the solutions developed, and (3) creation of exercises where users test and evaluate the TriPoD system under realistic conditions.

The research team shall, during pilot tests and exercises, investigate the attitudes, motivations, rules, and organizational issues surrounding interoperability and information sharing at all levels of MI management. Observations at training sessions and exercises will be performed, followed by focus group interviews. These will include 5-6 duty officers, 5-6 prehospital emergency care personnel with experience in prehospital MI management, and 5-6 hospital personnel with experience of in-hospital organization and management of an MI. All transcribed interviews and focus group interviews will be analyzed using systematic text condensation according to Malterud [45], guided by the COREQ (consolidated criteria for reporting qualitative research) checklist developed by Tong et al [46]. Malterud [45] describes 4 stages in the analysis process: (1) the text is read to capture themes, (2) the code units of meaning are extracted, (3) these are condensed into core content, and finally, (4) the findings are summarized. One researcher will be responsible and initiate the analysis, while the whole analysis process will be followed up by the research team to ensure trustworthiness. Questionnaires will be used to gain reflections of 10-15 emergency department physicians and nurses on real-time integrated information systems and the use of patient tags in the hospital setting.

#### Phase 4: Reflection

Reflections will be conducted at both individual and group levels. An important component of action research is reflection, where users’ ideas lead to modification of the technology solutions tested. Based on the results, the working group (n=10-12 health care professionals and technology producers) shall decide what to do next and modify the system prior to a new round of application and reassessment before implementation (Figure 1). Evaluation will focus on the end users’ opinion on TriPoD whether the technique meets the needs and expectations and will feature qualitative methods. A series of focus groups will be performed to solve the challenges and barriers identified during tests. These will include 5-6 duty officers, 5-6 prehospital emergency care personnel with experience of prehospital MI management, and 5-6 hospital personnel with expertise in hospital organization and management of an MI. Data will be analyzed using content analyses as described by Elo and Kyngäs [44]. The recruitment will be strategic to include participants with specific knowledge and experiences. The specific topic for this data collection is to keep the good changes, add new ones, and then begin the action research process again. This will continue until the working group is satisfied that the study objectives have been met. The outcomes of the cyclic process will be documented. In this action research study, a variety of data collection methods will be used [35,37,38,47], as displayed in Table 1.

**Table 1:** Description of the methods in the study process.

Phases	Methods	Sample size
Phase 1: planning	Workshops and structured meetings Literature review Semistructured interviews and questionnaires	10-12 health care professionals and technology producers 8 PECa personnel, 5-10 duty officers, and hospital personnel
Phase 2: action	Workshops Pilot tests/observations/field notes Reference group involvement	10-12 health care professionals and technology producers Prehospital and hospital settings 8-10 intended users
Phase 3: observation	Workshops and structured meetings Exercises/observations/field notes Focus group interviews Questionnaires	10-12 health care professionals and technology producers Command levels (regional/local) 5-6 duty officers, 5-6 PEC personnel, and 5-6 hospital personnel 10-15 emergency department personnel
Phase 4: reflection	Structured meetings Focus group interviews Reference group involvement Evaluation and analysis of total data material	10-12 health care professionals and technology producers 5-6 duty officers, 5-6 PEC personnel, and 5-6 hospital personnel 8-10 intended users

^a^PEC: prehospital emergency care.

These methods will be used to collect data prior to and during the development of technology and after the evaluation of the information technology developed. The intention is that the working group and intended users should come to a common understanding of how to solve real-life problems that do not have clear or obvious solutions during the developing process. Topics/questions to be covered are current practices, experiences in MI management, conventional methods versus new technologies, information needs, attitudes and expectations, real-time data transfer, interoperability, perceived feasibility and usefulness, perceived ease of use, and quality and safety issues.

### Data Analysis

As this study will have several data sources (workshops, focus group interviews, questionnaires, observations), it will provide a holistic perspective and limit the risk of bias when relying on limited data sources. The information obtained should have high scientific value and facilitate assessment of the system studied. The iterative approach enables discussions and analyses during ongoing data collection.

### Ethics Approval

This study has been approved by the Swedish Ethical Review Authority (Etikprövningsmyndigheten [EPM] 2023-04615-01; October 20, 2023), and in accordance with the guidelines of EPM, the research team will obtain informed written consent from the study participants. Participation is voluntary and may be withdrawn without consequences. Furthermore, confidentiality regarding interviews and observation data is guaranteed, and data will be stored securely.

## Results

The trial implementation and evaluation phase for this study is from April 2024 to May 2026. Based on meetings, interviews, and questionnaires in the planning phase, an early prototype has been developed. The findings from questionnaires in phase 1 gave valuable insights into users’ needs and wishes concerning communication and information sharing during an MI. Results from the full-scale exercise with figurants in phase 2 revealed that command centers received real-time updates on the patient count, triage, and position faster than traditional standard procedures of sharing information during an MI. Data transmitted through the web portal were updated each time a new patient was scanned by the prehospital emergency care personnel, allowing for continuous real-time monitoring and decision-making at command centers. Both prehospital emergency care personnel and the management personnel working at command centers observed TriPoD’s effectiveness and usability, despite minor delays when prehospital personnel on some cases failed in the efforts to consistently scan injured patients. We have performed observations in connection with 2 major exercises in April 2024 and November 2024. Final analyses of the observations will be reported. The outcome of the qualitative content analysis will form the basis for the design and development of the new information technology system. We aim to complete the observations in training sessions and exercises (phase 3) by September 2025, followed by modification of the technology solutions tested (phase 4) before dissemination in a scientific journal.

## Discussion

### Principal Findings

The main findings from this action study report will form the basis for the design and development process based on the experiences and requirements of end users. The main contribution of this study will be to assess the ways users work and to get their opinions on the functionality and usability of electronic devices. Different opinions are likely to arise on how information sharing can benefit from the implementation of a new technology, and this will enrich the project. However, it is not unusual that electronic devices are not used due to portability and usability issues [7]. If the device is impractical, it can significantly impact how successfully it is adopted in clinical practice and impede the implementation phase [5]. Despite decades of effort to create efficient information-sharing systems for communication between prehospital emergency care personnel, inefficiencies still persist [7]. Effective and timely exchange of information between command centers is crucial in the medical response to MIs and disasters [1-6], and any innovation in this field must be practical, user-friendly, and feasible [7]—not just the simple adoption of a new technique [33]. In a study by Reddick [5] on how communication technologies affect our readiness for MIs, results showed that technology is effective in emergency management, especially during the response phase.

### Comparison to Prior Work

TriPoD gives real-time information to command centers, supporting situational awareness by continuous update from the incident scene during transport and upon arrival at the hospital. TriPoD applies commercial technology and available information technology services in an innovative way. Although similar projects may be in progress, to our knowledge, no recommended system has been fully implemented at the time of this study.

### Strengths and Limitations

The strength of the methodology chosen is that it improves and changes practice by directly involving the intended users [38]. All methods used will be reported together with their justification so that other researchers can confirm the reliability of the study. However, it is challenging to replicate action research studies due to the context dependency, and the design offers explanatory theories that can be falsified. In addition, the research team consists of academicians from the field of emergency and disaster medicine, but there are no cocreating end users involved in the research process, and this can be considered as a limitation, as their perspectives will not be included in the research process. This approach requires trust between researchers, the working group, and the users involved in the development process of TriPoD and that all understand the value of rigor in research. Action research also has limitations since it usually involves very specific investigations, often within organizations, and findings can be considered to only apply to that setting. Although action research encourages the transferability of project outcomes, the generalizability of the findings is limited to context similarity [39]. The researchers are aware that the structure and quality of EMS can vary based on local, regional, national, and international context and resources. However, triage, delivery of emergency care, and transport to appropriate facilities are essential components of emergency care worldwide and enable the transferability of project outcomes to EMS in similar contexts.

### Dissemination Plan

Findings from this study plan will be disseminated through multiple channels to reach diverse audiences. Findings will be presented at national and international scientific conferences to engage with experts and practitioners in the field. Additionally, results will be published in peer-reviewed journals to ensure rigorous academic dissemination. We will also produce public scientific publications to make findings accessible to a broader audience, including practitioners and policy makers. To further enhance visibility, we will share the key results and updates on social media platforms, targeting both academic and professional communities. This strategy aims to increase the impact of our research by reaching relevant stakeholders.

### Future Directions

Locating patients can become complicated if multiple hospitals and adjacent regions are involved in an MI. After the implementation of TriPoD, a hypothesis is that the system can be further developed to support the reunification of family members. Furthermore, TriPoD is intended to increase civil-military synergies in the event of MI management.

### Conclusions

The result from the action research will highlight the ways intended users work, their opinions, and wishes to ensure high usability when developing and designing new technology in complex environments. Although the literature confirms the promising benefits of information technology, the opinions and needs of end users have yet to be addressed. Results will also support the practical implementation of new technology.

## Abbreviations

COREQ: consolidated criteria for reporting qualitative research
EMS: emergency medical services
EPM: Etikprövningsmyndigheten
MI: major incident
TriPoD: Triage, Position, and Documentation

## Data Availability

The datasets generated and analyzed during this study are available from the corresponding author on reasonable request.
